# Efficacy of Biogenic Zinc Oxide Nanoparticles in Treating Wastewater for Sustainable Wheat Cultivation

**DOI:** 10.3390/plants12173058

**Published:** 2023-08-25

**Authors:** Irfan Haidri, Muhammad Shahid, Sabir Hussain, Tanvir Shahzad, Faisal Mahmood, Muhammad Umair Hassan, Jameel Mohammed Al-Khayri, Mohammed Ibrahim Aldaej, Muhammad Naeem Sattar, Adel Abdel-Sabour Rezk, Mustafa Ibrahim Almaghasla, Wael Fathi Shehata

**Affiliations:** 1 Department of Environmental Sciences, Government College University, Faisalabad 38040, Pakistan; haidaryirfan807@gmail.com (I.H.); sabirghani@gmail.com (S.H.); hereistanvir@gmail.com (T.S.); 2 Department of Bioinformatics & Biotechnology, Government College University, Faisalabad 38040, Pakistan; mshahid@gcuf.edu.pk; 3 Research Center on Ecological Sciences, Jiangxi Agricultural University, Nanchang 330045, China; muhassanuaf@gmail.com; 4 Department of Agricultural Biotechnology, College of Agriculture and Food Sciences, King Faisal University, Al-Ahsa 31982, Saudi Arabia; maldaej@kfu.edu.sa (M.I.A.); arazk@kfu.edu.sa (A.A.-S.R.); wshehata@kfu.edu.sa (W.F.S.); 5 Central Laboratories, King Faisal University, P.O. Box 420, Al-Ahsa 31982, Saudi Arabia; mnsattar@kfu.edu.sa; 6 Department of Virus and Phytoplasma, Plant Pathology Institute, Agricultural Research Center, Giza 12619, Egypt; 7 Department of Arid Land Agriculture, College of Agriculture and Food Sciences, King Faisal University, Al-Ahsa 31982, Saudi Arabia; malmghaslah@kfu.edu.sa; 8 Plant Pests, and Diseases Unit, College of Agriculture and Food Sciences, King Faisal University, Al-Ahsa 31982, Saudi Arabia; 9 Plant Production Department, College of Environmental Agricultural Sciences, Arish University, North Sinai P.O. Box 45511, Egypt

**Keywords:** microbial synthesis, oxidative stress, antioxidants, photocatalytic degradation, municipal wastewater

## Abstract

Water scarcity due to overuse and growing water pollution has led to the need for upgrading of conventional methods of wastewater treatment. The biological synthesis of zinc oxide nanoparticles (ZnO-NPs) and their photocatalytic capacity to degrade contaminants offer a promising and environment-friendly approach to municipal wastewater treatment. This technique is advantageous due to its cost-effectiveness, sustainability, and reduction in toxic residual substances. In this study, microbial-synthesized ZnO-NPs were used for the treatment of municipal wastewater. The objective of this study was to evaluate the potential of treated wastewater for wheat crop cultivation. Zinc oxide nanoparticles were synthesized from a pre-isolated bacterial strain, namely *Shewanela* sp., and characterized using UV–VIS, X-ray diffraction (XRD), scanning electron microscopy (SEM), and Fourier transform infrared spectroscopy (FTIR) analyses. The results showed that after the treatment of wastewater, the concentration of total dissolve solids (TDS), the chemical oxygen demand (COD), and sulfate and phosphate levels decreased by 76.5%, 57.1%, 81.1%, and 67.4%, respectively. However, the application of treated wastewater increased chlorophyll, carotenoids, and antioxidants by 45%, 40.8%, and 10.5 to 30.6%, respectively. Further, the application of treated wastewater also significantly decreased oxidative stress induced by hydrogen peroxide (H_2_O_2_) and malondialdehyde (MDA) by 8.1% and 30.1%, respectively. In conclusion, biosynthesized ZnO-NPs could be an important choice to treat municipal wastewater and to improve wheat productivity.

## 1. Introduction

Water is an essential resource for maintaining the environment and fostering a sustainable ecosystem. Humans use water for drinking, sanitation, and many other activities [1]. The biological and chemical pollution of water has become the greatest issue confronted by the earth, especially during the past 30 years. Over the past three decades, nature has faced an escalating and pressing issue of biological and chemical pollution of water bodies [2,3]. The unregulated discharge of untreated wastewater has led to an increase in the concentration of potential toxic elements in the soil, thereby posing detrimental health problems for humans [3,4]. Many human pathogenic bacteria, like Vibrio cholerae, *Salmonella*, and *Shigella*, can be found in wastewater. Mercury and methyl mercury in wastewater disturb brain processes, leading to neurological diseases in human beings [3]. Biological pathogens, like *Leptospira*, *Shigella*, *Vibrio*, *Salmonella*, and *Leptospira*, cause various diseases in domestic and aquatic animals [4].

The contamination of water by volatile organic compounds (VOCs) and non-biodegradable heavy metals can hinder the use and recycling of water resources. There is a dire need to treat wastewater in an era of water shortage so that this wastewater can be used at least for agricultural purposes in agricultural fields. Traditional wastewater treatment methods include physical and chemical techniques [5,6]. In the twenty-first century, nanotechnology has emerged as a promising and rapidly advancing field of research. It addresses the existing limitations of conventional approaches and provides innovative nanoparticle-based approaches for wastewater treatment [7]. Nanotechnology is a more efficient and cost-effective method as compared to chemical and physical methods of wastewater treatment [5]. One of the notable advantages of nanotechnology is its potential to treat a large volume of wastewater, while using the minimal amount of energy and minimizing negative environmental impacts [8,9].

Recently, researchers have shown their interest in synthesizing different nanomaterials in order to meet the growing demand for efficient wastewater treatment [10]. Nanoparticles (NPs) are materials that typically have a size of 100 nm and display a wide range of shapes, such as spheres, rods, tubes, plates, stars, dots, wires, and prisms [11,12]. Due to their high reactivity and a larger surface-to-volume ratio, NPs possess special chemical, physical, optical, magnetic, and biological properties [9]. These characteristics of nanoparticles can make them vary in their size and form, making them highly versatile and suitable for a wide range of applications. They play a significant role in providing affordable removal of pollutants from wastewater using the adsorption process [13]. Nanoparticles have promising applications in different fields, but due to their unique properties and small size, they can be dispersed in ecosystems, potentially causing harm to living organisms throughout the food chain [11]. Moreover, the lack of thorough studies on the environmental impacts of NPs limits our ability to mitigate their potential adverse effects [12]. Metal oxide nanoparticles, including zinc oxide nanoparticles, have proven to be excellent adsorbents with high efficiency against various contaminants [14,15]. Zinc oxide exists in different crystalline forms, such as rock salt, wurtzite, and zinc blende, with a white or yellowish color. Researchers have shown keen interest in using zinc oxide nanoparticles for wastewater treatment due to their ability to dissolve in both acidic and basic solutions, as well as their higher stability in terms of charge and chemical bonding [16]. Taherizadeh and coauthors [17] reported that ZnO nanoparticles can adsorb and degrade pollutants from hospital waste, like phosphorus, resulting in a significant reduction in fouling. The mechanism of biogenic nanoparticle synthesis is based on the bioreduction of cations present in the precursor salts by amino acid enzymes and other biomolecules released by bacterial cells [16]. Mansour and coauthors [14] used green synthesis techniques to produce ZnO-NPs using red seaweed as the precursor salt. The efficiency of the synthesized ZnO-NPs as an adsorbent for IV2R dye removal was evaluated under various conditions. The study found that the maximum removal efficacy of IV2R reached 99% when 0.08 g of ZnO-NPs was used at a pH of 6, a temperature of 55 °C, and a contact time of 120 min. The dye adsorption capacity of the zinc oxide nanoparticles was determined to be 72.24 mg g^−1^, indicating a high potential for dye removal.

Various methods, including physical, biological, chemical, and hybrid methods, have been used for the synthesis of NPs [14,16]. Among them, the biological method is an affordable choice for the production of nanoparticles, such as silver, titanium, aluminum, nickel, and zinc oxide nanoparticles. The biological synthesis of nanoparticles is a preferable method due to its minimal energy consumption, the ability to generate non-toxic end products, and its eco-friendly nature [18,19]. Zinc acts as an essential nutrient for plants and has the ability to decrease the amount of non-essential elements, like cadmium, in the soil [15]. Zinc can be supplied to the soil–plant system in various forms to fulfill the nutritional requirements of plants. This study will offer a new approach to the biological and eco-friendly synthesis of ZnO-NPs from a bacterial strain, namely *Shewanela* sp., for efficient and sustainable agricultural practices under wastewater stresses. In this study, a green chemistry method was used for the synthesis of zinc oxide nanoparticles by using pre-isolated *Shewanela* sp. The synthesized nanoparticles were characterized and used to treat municipal wastewater due to their antibacterial properties, cost-effectiveness, sustainability, high reactivity, hydrophilicity, and lack of toxic by-products [20]. The wastewater treated with ZnO-NPs was then used to irrigate wheat crop in order to see the impact of the treated wastewater on the growth, physiological, antioxidant, and oxidant systems of wheat crop.

## 2. Materials and Methods

### 2.1. Synthesis of Zinc Oxide Nanoparticles (ZnO-NPs)

The pre-isolated bacterial strain *Shewanela* sp. was used to synthesize zinc oxide nanoparticles. This strain was obtained from the Environmental Microbiology Lab, Government College University Faisalabad (31.4° N, 73.06° E). *Shewanela* sp. cells were cultured in a broth medium using distilled water for 24 h. Afterward, 25 mL of the culture was extracted and mixed with 75 mL of distilled water to create a diluted culture. This diluted culture was then allowed to grow for an additional 24 h. After 24 h, 10 mM of ZnSO_4_·7H_2_O (Sigma-Aldrich, CAS number: 7446-20-0) was added to the culture, and the culture was placed in a shaking incubator at 37 rpm for the next 24 h until a white deposit was formed at the bottom of the conical flask, indicating ion transformation. After cooling, the culture fluid was incubated at room temperature, and visible white clusters coalesced and settled at the flask’s bottom within 12–48 h of rotary shaker incubation. The broth culture was then centrifuged at 3700 rpm for 15 min after incubation [21].

### 2.2. Characterization of Zinc Oxide Nanoparticles

In order to characterize zinc oxide nanoparticles, the suspension was centrifuged at a high speed of 12,000 rpm. The resulting pellet was dried using lyophilization, yielding a powder of nanoparticles, which was then used for characterization and further applications. UV–VIS spectroscopy (STA-8200V STALWART Van Nuys USA) was used to determine the presence of nanoparticles by scanning between the range of 300 nm and 600 nm.

The structural crystallinity and average size of zinc oxide nanoparticles were manually analyzed using X-ray diffraction. In this process, X-rays were irradiated onto the nanoparticles, and then the intensity and angle of scattering of the rays passing through the nanoparticles were measured. A scientific thermo-diffractometer (PANalytical X’PERTPRO, USA) was used to analyze the X-ray diffraction of zinc oxide nanoparticles. The average size of zinc oxide nanoparticles was determined using the Debye–Scherer formula D = Kλ/β cosθ, where k = shape factor, λ = wavelength of radiation, and D = size of the crystallite [22]. Fourier transform infrared spectroscopy (FTIR) spectra were confirmed using the PerkinElmer Spectrum-100 FT-IR spectrometer (FTIR-Bruker TENSOR-27). The distinctive functional groups and related proteins present on the surfaces of the biologically produced nanoparticles were confirmed by setting the range between 4000 and 400 cm^−1^. For this process, 1 g of the sample was taken and placed on a small crystal area against the high-refractive-index prism [23].

Scanning electron microscopy (SEM LEO 1530, Germany) was used to determine the surface morphology of nanoparticles, following the procedure outlined by Awasthi and coauthors [19]. The surface morphology and shape of ZnO-NPs were examined using SEM [24], which generated high-magnification images by directing a beam of electrons onto the nanoparticles. A solution of highly dispersed zinc oxide nanoparticles was coated onto a silicon chip and allowed to completely dry before placing it for morphology determination.

### 2.3. Application of Zinc Oxide Nanoparticles for the Treatment of Wastewater

Municipal wastewater was collected from the Nalka Kohala Sargodha Road, Faisalabad, Pakistan (31.30° N and 73.04° E). Particle matter was removed from the wastewater samples by centrifuging them at 10,000 rpm for 5 min. In order to assess the efficiency of biosynthesized ZnO-NPs, 1 g of NPs was added to 1000 mL of wastewater, while maintaining the control without nanoparticles [25,26]. The mixture was vortexed and then incubated for approximately 7 h in sunlight. After the treatment of wastewater, the sample was centrifuged for 10 min at 10,000 rpm to remove the zinc oxide nanoparticles (ZnO-NPs). The pH, electrical conductivity, and total dissolved solids were measured by following the previously described standard methods for the examination of water and wastewater by Rice and coauthors [25]. The chemical oxygen demand of wastewater was determined by taking 50 mL of the sample and then adding 5 mL of concentrated sulfuric acid, 1 g of mercury sulfate, 25 mL of potassium dichromate, and 70 mL of sulfuric acid to the sample. This solution was heated for 2 h and then cooled at room temperature, and then six to eight drops of a ferron indicator were added to the sample. The digesting solution was titrated with 0.25 N ferrous ammonium sulfate [25].

The gravimetric technique was used to analyze sulfates by taking 25 mL of the wastewater sample, to which 1 ml of HCL was added [1]. Subsequently, 5 mL of BaCl2 solution was added and vigorously shaken for 1 or 2 min. Standard solutions were prepared using potassium sulfate with concentration ranges from 10 to 200 ppm. The sulfate concentration was determined by measuring absorbance in a spectrophotometer (STA-8200V STALWART Van Nuys USA) at a wavelength of 420 nm, along with the standard solutions. Phosphate in the wastewater was determined by using colorimetric techniques. For this purpose, 25 mL of the sample was taken, and the pH of the sample was adjusted using 5N H_2_SO_4_ and NaCl solution. Standard phosphate solutions were prepared ranging from 1 to 5 ppm, and two blank solutions were prepared using distilled water. The absorbance of the solution was determined using a spectrophotometer at a wavelength of 882 nm. The color intensity was determined using an ultraviolet spectrophotometer (STA-8200V STALWART Van Nuys USA) over a range of 400–700 nm [2]. The decolorization activity (%) was measured with the following formula:

Decolorization = [(Absorbance of control sample − Absorbance of test sample)/Absorbance of control sample] × 100%(1)

### 2.4. Application of Treated Wastewater for Cultivation of Wheat Crop

#### Pot Experiment

For the cultivation of wheat, seeds were disinfected with 5% *w*/*v* sodium hypochlorite for 10 min, followed by five washes with sterile water. Soil collected from the Ayub Agriculture Research Institute was used to grow wheat crop in pots. The physiochemical properties of the soil included a clay loam texture, a pH of 7.6, an electrical conductivity of 2.5 dSm^−1^, a nitrogen content of 0.61%, a phosphorus concentration of 12.8 ppm, a potassium level of 154 ppm, and an organic matter content of 0.74%. Each pot was filled with 500 g of soil. Treated and untreated wastewater was separately applied to the pots. After 3 days of irrigation with untreated and treated wastewater, sterilized seeds were planted, with 10 seeds per pot. Following seed germination, treated and untreated wastewater was regularly applied separately until crop harvesting. Distilled water was used as the control. The plant seedlings were irrigated alternatively with treated and untreated wastewater treatments using 0.5X Hoagland solution for a period of 30 days. The germination percentage was determined at days 3, 6, and 9 [26]. The experiment was carried out in triplicate, and the statistical completely randomized design (CRD) was used. The experiment was conducted in a growth chamber having a temperature range of 15−25 °C and a humidity level of 65% with 7 h of photoperiod.

### 2.5. Determination of Plant Morphological Parameters

Wheat plants were harvested 30 days after sowing. Plants samples were cleaned to remove debris, and morphological parameters were measured by taking average plants from respective treatments. Shoot lengths and root lengths were measured in centimeters using a measuring tape, while shoot weights and root weights were measured in grams using a weighing balance. The roots were then dried on filter paper after being rinsed with distilled water. The fresh weights of the roots and shoots were then measured in grams using a weighing balance. Roots and shoots were placed in an oven for 48 h at 100 °C to estimate dry weights [27].

#### 2.5.1. Pigment Content Assay

The chlorophyll content, including chlorophyll a, chlorophyll b, total chlorophyll, and carotenoids, was evaluated using the protocol developed by Armon [28]. About 0.1 g of a fresh leaf sample was ground with 80% acetone (*v*/*v*) along with liquid nitrogen to prevent enzyme denaturation. The ground sample was then centrifuged at 12,000 rpm, and the supernatant was collected and stored in a refrigerator at 4 °C. Next, the enzyme extract was centrifuged for 10 min at 10,000 rpm. At three different wavelengths of 645, 480, and 663 nm, the absorbance of the supernatant was recorded, respectively, for chlorophyll a, chlorophyll b, and carotenoids using a spectrophotometer (STA-8200 V STALWART Van Nuys USA). The chlorophyll content was calculated using the given formula [29].

Chlorophyll a = {12.7 (OD663 − 2.69 (OD645) × V/10,000 × W}(2)

Chlorophyll b = {22.9 (OD645 − 4.68 (OD663) × V/10,000 × W}(3)

Total Chl. = [20.2 (OD 645) − 8.02 (OD 663)] × V/W × 1/10,000(4)

A car = µg/g FW- = OD 480 + (0.114 × OD 663) × (0.638 × OD 645)(5)

where W = weight of the fresh leaf tissue (g), V = volume of the extract (mL), Car = A Car/Em 100% × 100, emission = Em 100% = 2500, and OD = absorbance at the respective wavelength.

#### 2.5.2. Antioxidants

The phenolic content of fresh plant leaves was determined by grinding 0.5 g of leaves in 80% (*v*/*v*) acetone with 5 mL of the enzyme extract. The resulting reaction mixture was centrifuged for exactly 10 min at a speed of 10,000 rpm. Next, 2 mL of distilled water (DW), 1 mL of the supernatant, and 1 mL of Folin–Ciocalteu phenol reagent was added to the solution. After shaking the reaction mixture, 20% sodium carbonate was added to the reaction mixture to make the volume of the reaction mixture up to 10 mL using distilled water. The absorbance of the reaction mixture was measured at a wavelength of 750 nm using a spectrophotometer [30]. Ascorbate peroxidase (APX) activity was determined by using the method of Amako and coauthors [31]. For this purpose, 1000 µL of the reaction mixture was prepared by adding 700 µL of phosphate buffer, 100 µL of 0.5 mM ascorbate, and 100 µL of enzyme extract. The absorption of the mixture was measured using a spectrophotometer (STA-8200 V STALWART Van Nuys USA) at a wavelength of 290 nm after every 20 s.

In order to determine catalase (CAT) activity, centrifugation was carried out at 10,000 rpm for 10 min after crushing leaf tissues with phosphate buffer (50 mM and pH 7.8), following the procedure outlined by Aebi and coauthors [32]. The samples were homogenized with 0.75 mM of H_2_O_2_. A UV–VIS spectrophotometer (STA-8200 V UV/Vis STALWART Van Nuys USA) was used to measure the decrease in absorbance at 240 nm. Peroxidase activity (POD) was measured by following the method of Chance and Maehly [33]. Plant tissues were homogenized in phosphate buffer (50 mM, pH 7.8) and then centrifuged at 8000 rpm at 25 °C. For the estimation of superoxide dismutase (SOD), the methodology of Giannopolitis and Ries [34] was adopted. A 3 mL reaction solution was prepared by combining 50 µL of riboflavin, 50 µL of nitro blue tetrazolium (NBT), 100 µL of L-methionine, 50 µL of enzyme extract, 100 µL of triton-X, and 400 µL of H_2_O. After mixing them, the reaction mixture was put in a test tube, and readings were taken using a UV–VIS spectrophotometer (STA-8200V STALWART Van Nuys USA) at a wavelength of 560 nm.

#### 2.5.3. Oxidative Parameters

The Heath and Packer [35] method was adopted to measure the malondialdehyde (MDA) content of plant fresh shoots using thiobarbituric acid (TBA). Trichloroacetic acid (TCA) (0.1%) was used to grind the shoots. After centrifugation of the mixture, the supernatant was collected. TCA (20%) along with TBA (0.5%) was added, and the mixture was heated at 100 °C for approximately 30 min. After cooling, the mixture was centrifuged again, and the resulting supernatant was analyzed using a spectrophotometer (STA-8200 V STALWART) to determine absorbance at 532 nm [27]. The Jana and Choudhuri [36] method was used to calculate the peroxidase (H_2_O_2_) concentration of the plants. Fresh plant samples (0.1 g) were ground in an ice tub with 5 mL of 0.1% (*w*/*v*) trichloroacetic acid. Next, 1 mL of KI was added to 0.5 mL of potassium phosphate buffer; subsequently, 0.5 mL of the supernatant was added. The sample was centrifuged and left at room temperature for 10 min. Absorbance was measured at 390 nm using a spectrophotometer (STA-8200 V STALWART). The H_2_O_2_ content was determined by comparing with the standard curve generated using distinct H_2_O_2_ concentrations. The H_2_O_2_ values were determined in terms of µmol/g FW.

### 2.6. Statistical Analysis

One-way analysis of variance (ANOVA) was performed using Statistix (version 8.1) software to analyze the data and the least significant difference (LSD) test at a 95% confidence level to determine the difference between the treatments.

## 3. Results

### 3.1. Characterization of Zinc Oxide Nanoparticles

**Figure 1:**
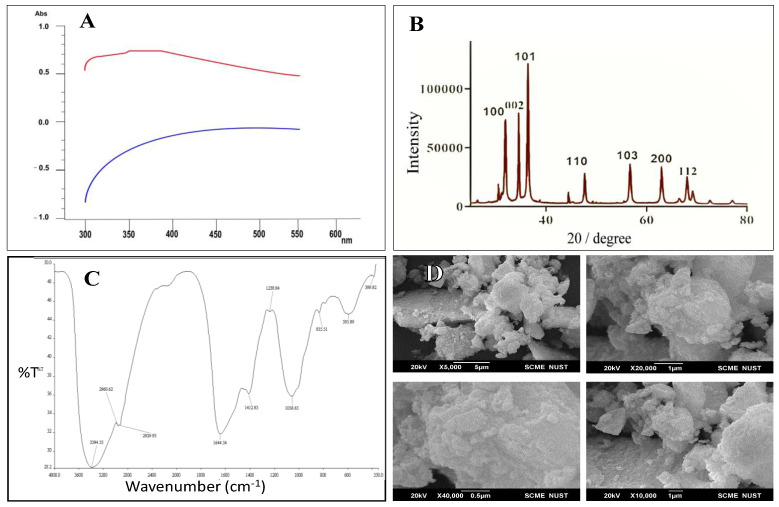
Characterization of ZnO-NPs: UV–VIS spectroscopy (**A**), XRD (**B**), FTIR (**C**), and SEM (**D**).

After adding salt to the culture media, the color of the supernatant changed from green to yellowish within 24 h. The culture media were subsequently subjected to UV–VIS spectroscopy, revealing the formation of a peak ranging from 200 nm to 600 nm. Figure 1A displays a prominent absorption peak between 360 nm and 380 nm within the 200–600 nm range, confirming the successful synthesis of zinc oxide nanoparticles. X-ray diffraction analysis was carried out on the powdered form of the nanoparticles using a diffractometer instrument, with scanning performed between 100 nm and 800 nm. The diffraction peaks at 100, 002, and 101 of XRD analysis of ZnO-NPs confirmed the crystalline structure with an average size of 50 nm at the 200 peak (Figure 1B). The FTIR spectrum of the synthesized nanoparticles was obtained within a wave number range of 4000–400 cm^−1^ up to 40 cycles. The FTIR spectrum of ZnO-NPs showed broad peaks at 3394 cm^−1^, 1644 cm^−1^, 1412 cm^−1^, and 1056 cm^−1^ (Figure 1C). The peaks at 3394 cm^−1^ and 1644 cm^−1^ confirmed the presence of OH and carbonyl groups, while the peaks at 1412 and 1056 cm^−1^ confirmed the C-N and C-O stretch. Field emission SEM was used to determine the shape, morphology, and elemental composition. The nanoparticles exhibited a slightly spherical-to-irregular shape, with diameters ranging from 21 nm to 47 nm (Figure 1D).

### 3.2. Treatment of Wastewater

Table 1 presents the preliminary characterization of municipal wastewater collected from the Nalka Kohala Sargodha Road, Faisalabad. The results indicated that the pH, EC, TDS, color intensity, COD, sulfates, and phosphates were 8.7, 2.39 dS/m, 3860 mg L^−1^, 0.253, 336.5 mg L^−1^, 276.4 mg L^−1^, and 2110 mg L^−1^, respectively, in the municipal wastewater. According to the National Environmental Quality Standards (NEQS) set by the Government of Pakistan, the levels of these parameters in the municipal wastewater exceed the normal limits. Therefore, ZnO-NPs were used for treating the municipal wastewater. After treatment of wastewater, the pH, EC, TDS, color intensity, COD, sulfates, and phosphates were reduced significantly by 11.1%, 48.1%, 76.4%, 64.4%, 57%, 81%, and 67%, respectively, in treated wastewater as compared to untreated wastewater, as shown in Table 1.

### 3.3. Physical Growth Parameters of Wheat Crop

The wheat crop irrigated with treated wastewater showed significant enhancement in the physical growth parameters of wheat compared to untreated-wastewater-irrigated crop (Table 2). In the pot experiment, the shoot and root lengths of the wheat crop irrigated with treated wastewater were significantly higher (*p* ≤ 0.05), showing an increase of 30.8% and 38.8%, respectively, in comparison to the wheat crop irrigated with untreated wastewater. Nevertheless, these lengths were slightly lower, by 2% and 6.34%, respectively, than those of the wheat crop irrigated with distilled water. Similarly, compared to untreated wastewater, the application of treated wastewater significantly increased both shoot fresh weights and root fresh weights, reaching up to 59.7% and 46%, respectively. However, these values were found to be 8.33% and 16.15% lower, respectively, than those observed with distilled wastewater. Similarly, the shoot and root dry weights increased by 41.75% and 65.7% in treated wastewater as compared to non-treated wastewater. In contrast, when exposed to distilled water, these percentages increased by 7% and 12.71%.

It is concluded that wheat crop irrigated with treated wastewater exhibits significantly greater growth compared to non-treated crop; however, the disparity in growth between the wheat crop irrigated with treated wastewater and with distilled water (controls) was not statistically significant (Table 2).

### 3.4. Germination Rate

**Figure 2:**
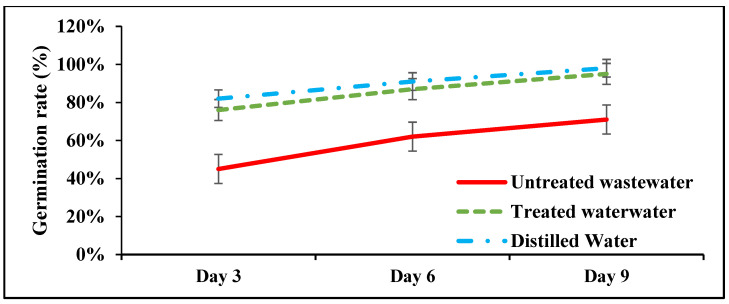
Effects of ZnO-NP-treated wastewater, untreated wastewater, and distilled water on the germination rate of wheat noticed at days 3, 6, and 9. Data are means + SD (*n* = 3).

The wastewater treated with ZnO-NPs significantly (*p* < 0.05) increased the germination rate of wheat crop on days 3, 6, and 9 compared to untreated wastewater. It was observed that on days 3, 6, and 9, the germination rate in the treated wastewater increased by 45%, 31%, and 27.5%, respectively. However, in distilled wastewater, the germination rate slightly increased by 7.32%, 4.4, and 3.06%, respectively, compared to the treated wastewater plants; these differences were statistically non-significant (Figure 2).

### 3.5. Chlorophyll Content

**Figure 3:**
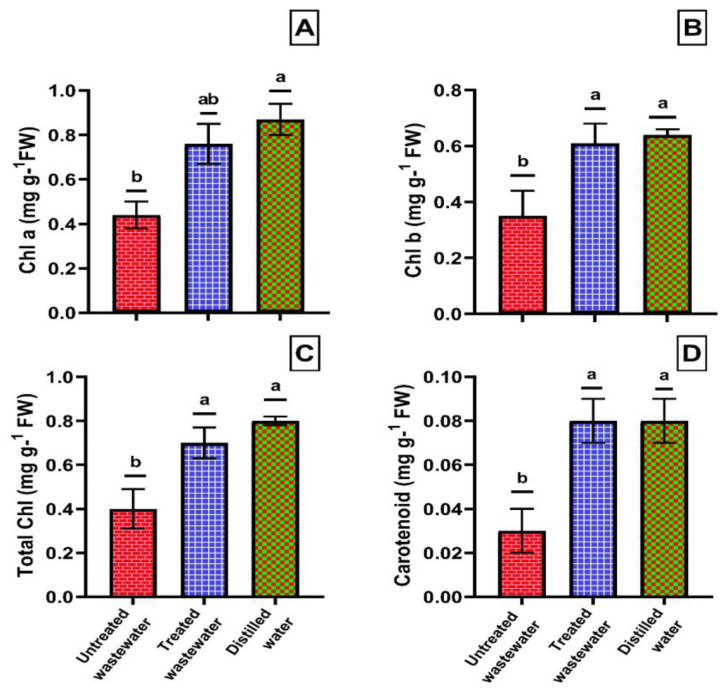
Effects of treated wastewater, untreated wastewater, and distilled water on changes in the content of chlorophyll a (**A**), chlorophyll b (**B**), total chlorophyll (**C**), and carotenoids (**D**) of wheat. Data are means + SD (*n* = 3). Different small letters at different parameters indicate a significant difference between parameters at *p* < 0.05.

The treated wastewater showed positive effects on the chlorophyll content of wheat crop. The application of treated wastewater led to a significant increase in the concentration of chlorophyll a and b by 42.6% and 42.9%, respectively, as compared to untreated wastewater. However, when compared to distilled water, there was a slight increase of 12.57% and 4.07%, respectively, which was statistically non-significant. A similar pattern was observed for the carotenoid contents, which was 67.39% higher in treated wastewater compared to non-treated wastewater. In the case of distilled water application, the carotenoid content increased by 0.3% as compared to treated wastewater, which was statistically at par with each other (Figure 3A–D).

### 3.6. Activity of Oxidants and Antioxidants

**Figure 4:**
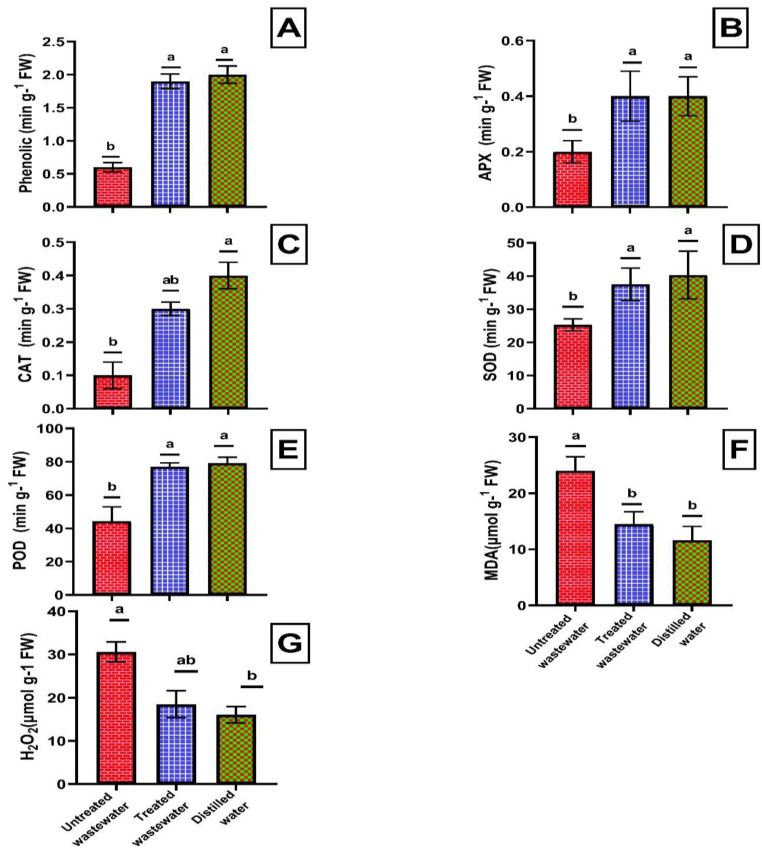
Effects of treated wastewater, untreated wastewater, and distilled water on changes in the content of phenolics (**A**), APX (**B**), CAT (**C**), SOD (**D**), POD (**E**), MDA (**F**), and H_2_O_2_ (**G**) of wheat crop. Data are means + SD. Different small letters at different parameters indicate a significant difference between parameters at *p* < 0.05.

The wastewater treated with ZnO-NPs improved the antioxidant system of wheat crop. The concentrations of phenolics, APX, CAT, SOD, and POD showed significant increases (*p* ≤ 0.05) of 69.3% 36.40% 56.9%, 40.3%, and 42.3%, respectively, in wheat plants irrigated with treated wastewater compared to those irrigated with untreated wastewater. Similarly phenolic, APX, CAT, SOD, and POD contents were 2.09% 14.22%, 16.01%, 6.9%, and 2.58% lower, respectively, in wheat crops irrigated with treated wastewater compared to those irrigated with distilled water. However, statistical analysis indicated that these differences were not significant (Figure 4A–E). The treated wastewater reduced the concentration of MDA and H_2_O_2_ by 65.12% and 65.72%, respectively, in the wheat crop compared to untreated wastewater. However, in distilled water, this reduction was slightly greater, with a decrease of 24.90% and 14.58%, respectively, as compared to treated wastewater (Figure 4F,G).

## 4. Discussion

The direct discharge of municipal wastewater into the environment without treatment is one of the major issues being faced by cities, especially agricultural areas [37]. Nanoparticles could potentially play a crucial role in treating municipal wastewater before its discharge into the natural environment. Many nanoparticles are used to treat municipal wastewater, but ZnO-NPs are particularly well suited for the treatment of municipal wastewater, owing to their antibacterial properties, which effectively eliminate pathogens present in the municipal wastewater [38]. Zinc oxide is regarded as environmentally friendly due to its non-toxic and biocompatible nature. The nanoparticles can be easily separated and recovered after treatment, thereby minimizing the risk of environmental contamination [39].

ZnO-NPs were synthesized biologically because biosynthesized nanoparticles are eco-friendly, safe, easy, and non-toxic [40]. *Shewanela* sp. was used as a microbial source for synthesizing zinc oxide nanoparticles. In comparison to plant-based synthesis, the microbial synthesis of nanoparticles offers a more efficient, manageable, and flexible mechanism for nanoparticle production [41]. The optical properties of biosynthesized ZnO-NPs were determined using UV–VIS absorption spectroscopy. The absorption spectra of ZnO-NPs illustrated the maximum absorption between 360 to 380 nm due to the transmission of electrons from the valance band to the conduction band. The XRD analysis findings, presented in Figure 1B, demonstrated diffraction peaks at 100, 002, and 101, indicating the crystalline structure of ZnO-NPs. The average size of the nanoparticles was determined to be 50 nm. These results are aligned with previous research conducted by Abdullah and coauthors [42] and previous studies [41,42]. The results given in Figure 1C illustrate the FTIR spectrum of ZnO-NPs with broad peaks at 3394 cm^−1^, 1644 cm^−1^, 1412 cm^−1^, and 1056 cm^−1^. The peaks at 3394 cm^−1^ and 1644 cm^−1^ confirmed OH and carbonyl groups, which is according to previous findings [43]. Rambabu and coauthors [44] demonstrated a similar result for the FTIR spectrum of ZnO-NPs, where the peak at 3379 cm^−1^ was attributed to the −OH stretch. The peaks at 1412 and 1056 cm^−1^ confirmed the C-N and C-O stretch. These peaks were also observed by Ifeanyinchukwa [45], where 1053 cm^−1^ and 1352 cm^−1^ represented the C-O and C-N stretch. Using scanning electron microscopy, the morphology of ZnO-NPs was determined, as shown in Figure 1D. The results showed that ZnO-NPs have a slightly spherical-to-irregular shape within a diameter range of 21–47 nm. Similar results for zinc oxide nanoparticles have also been reported by previous studies [42].

The biosynthesized ZnO-NPs were used at a concentration of 1 g/L for treating contaminants from municipal wastewater, as reported in previous studies [46]. These results were also in accordance with those of Puay and coauthors [47], in which they used ZnO-NPs at a concentration of 1 mg/L for the treatment of wastewater. However, this result was correlated with that of Soltani and coauthors’ [48] study, where they used 3 mg of ZnO-NPs for the degradation of 200 mL of MB dye. ZnO-NPs play a crucial role in the decolorization and degradation of pollutants in wastewater. When exposed to light, they generate electron–hole pairs, which subsequently interact with oxygen and water. This interaction leads to the formation of highly reactive species, such as hydroxyl radicals [49]. These generated free radicals might interact with the contaminants and transform them into less toxic and simpler molecules [17,49,50].

The decrease in the pH and EC of treated wastewater might be due to the breakdown of organic matter present in the municipal wastewater through oxidation or other chemical process. Furthermore, this decline might also be due to the fact that ZnO-NPs precipitate dissolved ions, especially those that form insoluble carbonate or hydroxide compounds in an alkaline environment [51,52]. The total dissolved solids (TDS) were removed significantly (*p* < 0.05) by up to 76.42%. These results are in accordance with Taherizadeh and coauthors [17], where they found a 100% reduction in the TDS by using ZnO nanoparticles for wastewater treatment. The TDS removal from wastewater might be because of a process such as adsorption due to its unique properties, including a high surface-area-to-volume ratio and a large number of active sites for interacting with the TDS [51]. ZnO-NPs might precipitate TDS ions by offering active nucleation sites, transforming them into solid precipitates [52]. Total dissolved solids can also be eliminated through photocatalytic degradation, facilitated by the generation of reactive oxygen species in the presence of sunlight. These reactive oxygen species oxidize and subsequently break down the TDS ions, converting them into less harmful substances [53]. Sulfates and phosphates were removed by up to 81.04% and 76.64%, respectively, with treated wastewater as compared to untreated wastewater (Table 1). These results are consistent with Taherizadeh and coauthors [51], where the phosphate value was reduced by 99% when they treated wastewater with ZnO-NPs. A similar study was also conducted by Taherizadeh and coauthors [16] using 1 mg/L of ZnO-NPs for the treatment of municipal wastewater, resulting in a significant reduction in the concentrations of phosphorus, sulfates, and other pollutants. The color intensity and COD reduced by 64.43% and 57.08%, respectively, in treated wastewater as compared to untreated wastewater. These results are consistent with previous studies [54]. A similar study was conducted by Soltani and coauthors, where ZnO-NPs were synthesized using Scallion’s peel extract and used for the degradation of MB dye. The maximum MB dye removal achieved was 97% after 1 h and 25 min using a CuO/ZnO20/80 nanocomposite. ZnO-NPs decolorize and degrade pollutants in wastewater by producing electron–hole pairs when they are exposed to light. These pairs subsequently interact with oxygen and water, leading to the formation of highly reactive species, such as hydroxyl radicals. As these radicals interact with the contaminants, they can transform them into less toxic and simpler molecules [50,55].

Wastewater disturbs the physical and physicochemical systems of wheat due to the high concentration of COD, TDS, and other nutrients, like sulfates and phosphates. The high amsount of phosphates and nitrates might disturb the metabolism by unbalancing the nutritional system of the plant and can accumulate in cells and tissues [17]. Physical growth parameters of wheat decreased considerably in untreated wastewater as compared to treated wastewater (Table 2). The presence of pollutants in wastewater may lead to a reduction in oxygen availability by contaminating the soil around wheat. As a result, this can retard the physical growth of wheat plants [56]. Results showed that the lengths, weights, chlorophyll contents, and carotenoid contents of wheat in untreated wastewater decreased significantly as compared to treated wastewater. Based on our observations, we did not find any studies on the applications of zinc-oxide-nanoparticle-treated wastewater used in the cultivation of wheat crop. However, Kanwal and coauthors grew wheat in wastewater, and their results demonstrated that wastewater has adverse effects on the plant height (by 33%), number of leaves (by 41%), root fresh weight (by 50%), shoot fresh weight (by 62%), root dry weight (by 63%), shoot dry weight (by 71%), and root length (by 45%). Moreover, physiological parameters, such as stomatal conductance (by 82%), transpiration rate (by 72%), and photosynthetic rate (by 74%), were negatively affected. The reduction in growth might be due to the presence of a high number of salts, such as phosphates and nitrates, which lead to salinization [57]. In a study conducted by Kanwal and coauthors [58], wheat was cultivated using untreated wastewater, and the results showed a decrease of 42% in chlorophyll a, 53% in chlorophyll b, and 41% in carotenoids.

The activity of SOD, POD, CAT, APX, and phenols in wheat in non-treated wastewater decreased as compared to treated wastewater, while oxidants, like MDA and H_2_O_2_, increased in untreated wastewater as compared to treated wastewater. The presence of residual chemical and toxic substances in untreated wastewater might generate free radicals; these free radicals are reactive in nature and can damage plant tissues. These free radicals can elevate the level of oxidants within plants, thus leading to oxidative stress [55]. A similar study was conducted by Sen and coauthors [59], where bioreactor-treated textile wastewater led to increased carbohydrate (241%) and protein (212%) as compared to untreated wastewater. This study is unique because according to the best of our knowledge and observation, there is no single study on the production of zinc oxide nanoparticles using *Shewanela* sp. for the treatment of municipal wastewater and the decrease in the stress of municipal wastewater on wheat crop. This research holds significance as it introduces a novel biological approach to ZnO-NP synthesis and offers a sustainable solution for municipal wastewater treatment, thus conserving dwindling water resources for agricultural use. There might be a possibility that some ZnO-NPs could remain in the treated wastewater after centrifugation due to carelessness or instrumental inaccuracy. These residual concentrations of ZnO-NPs in treated wastewater could be harmful due to their antimicrobial properties. These antimicrobial properties could potentially harm aquatic ecosystems by disrupting the normal metabolic functions of aquatic organisms and altering microbial communities [60]. ZnO-NPs, when accumulated in the food chain, could lead to bioaccumulation, disturbing the health of different trophic levels. Properties such as the persistence of ZnO-NPs in water could result in long-term ecological disturbances and disruptions of ecosystem services. Therefore, careful handling and further research are required to mitigate these potentially harmful outcomes [60].

## 5. Conclusions

The study highlights the potential of biologically synthesized zinc oxide nanoparticles (ZnO-NPs) as an efficient and sustainable method for treating municipal wastewater. The treatment of wastewater with biologically synthesized ZnO-NPs is a non-toxic, cost-effective, and sustainable process. The treatment of wastewater using 1 g/L of ZnO-NPs resulted in substantial reductions in the EC and color intensity and in the concentrations of TDS, COD, sulfates, and phosphates by 48.12%, 64.43%, 89.90%, 57.08%, 81.04%, 76.64%, and 38.46%, respectively. Moreover, the treated wastewater exhibited positive effects on wheat crop by promoting the growth and germination rates. The chlorophyll content and enzymatic activity in wheat crop increased, indicating efficient physiological functions of the crop. These findings emphasize the cost-effectiveness, sustainability, and potential environmental benefits of using biologically synthesized ZnO-NPs for wastewater treatment. Effectively reducing the amounts of contaminants and the phytotoxicity of the treated wastewater, this approach is a promising tool for addressing water pollution concerns and supporting agricultural productivity. This research holds significant importance in evaluating the environmental safety and sustainability of ZnO-NP-based wastewater treatment methods, contributing to informed decision making and regulatory measures in ensuring the protection of ecosystems and public health. By using advanced molecular and biochemical techniques, such as gene expression profiling and oxidative stress markers, the extent of toxicity, potential accumulation, and long-term effects of ZnO-NPs on various aquatic life forms and the potential implications for human health can be elucidated.

## Figures and Tables

**Table 1:** Pollutants decreased in municipal wastewater after ZnO-NP treatment and comparison with the NEQS.

Parameters	Wastewater	Treated Wastewater	NEQS Limits	% Decrease
pH	8.7 ± 0.10	7.73 ± 0.31	6.5–8.5	11.1%
EC (ds m^−1^)	2.39 ± 0.02	1.24 ± 0.10	NG	48.1%
TDS (mg/L)	3860 ± 16	910 ± 12.06	1000	76.4%
Color intensity	0.253 ± 0.02	0.09 ± 0.02	NG	64.4%
COD (mg/L)	336.5± 7.25	144.43 ± 5.7	200	57%
Sulfates (mg/L)	276.4 ± 6.05	52.4 ± 6.81	500 mg/L	81%
Phosphates (mg/L)	2110 ± 5.9	690 ± 15.1	NG	67.3%

Each value presented is the mean of three replicates, accompanied by the standard deviation. It is important to note that the National Environmental Quality Standards (NEQS) of Pakistan govern the permissible limits of wastewater for irrigational purposes. NG: not given.

**Table 2:** Effects of treated wastewater, untreated wastewater, and distilled water on physical parameters of wheat plants.

Treatment	Shoot L (cm)	Root L (cm)	Shoot FW (g)	Root FW (g)	Shoot DW (g)	Root DW (g)
Untreated wastewater	12.4 ± 1.11 ^b^	7.3 ± 0.90 ^b^	0.21 ± 0.03 ^e^	0.24 ± 0.12 ^b^	0.04 ± 0.02 ^b^	0.06 ± 0.03 ^b^
Treated wastewater	17.9 ± 1.74 ^a^	11.9 ± 2.07 ^a^	0.51 ± 0.07 ^ab^	0.45 ± 0.13 ^ab^	0.07 ± 0.02 ^a^	0.18 ± 0.06 ^a^
Distilled water	18.3 ± 1.68 ^a^	12.7 ± 2.29 ^a^	0.56 ± 0.06 ^a^	0.54 ± 0.13 ^a^	0.08 ± 0.02 ^a^	0.20 ± 0.08 ^a^

Data are means + SD (*n* = 3). Different small letters at different parameters indicate a significant difference between parameters at *p* < 0.05.

## Data Availability

Not applicable.
